# A simple way to evaluate self-designed probes for tumor specific Multiplex Ligation-dependent Probe Amplification (MLPA)

**DOI:** 10.1186/1756-0500-3-179

**Published:** 2010-06-26

**Authors:** Kristina Pedersen, Emilia Wiechec, Bo E Madsen, Jens Overgaard, Lise Lotte Hansen

**Affiliations:** 1 Institute of Human Genetics, The Bartholin building, Wilhelm Meyers Allé 4, University of Aarhus, DK-8000 Aarhus C, Denmark; 2 Department of Experimental Clinical Oncology, Aarhus University Hospital, Nørrebrogade 44, DK-8000 Aarhus C, Denmark; 3 Bioinformatics Research Center (BiRC), University of Aarhus, DK-8000 Aarhus C, Denmark; 4 Greenbaum Cancer Center, University of Maryland, 655 West Baltimore Street; 21201 Baltimore, MD, USA; 5 AgroTech, Institute for Agri Technology and Food Innovation, Udkærsvej 15, 8200 Aarhus N, Denmark

## Abstract

**Background:**

The Multiplex Ligation-dependent Probe Amplification (MLPA) is widely used for analysis of copy number variations (CNVs) in single or multiple loci. MLPA is a versatile methodology and important tool in cancer research; it provides precise information on increased or decreased copy number at specific loci as opposed to loss of heterozygosity (LOH) studies based upon microsatellite analysis. Pre-designed MLPA kits and software are commercially available to analyze multiple exons, genes, and genomic regions. However, an increasing demand for new gene specific assays makes it necessary to self-design new MLPA probes for which the available software may not be applicable. During evaluation of new self-designed reference probes, we encountered a number of problems, especially when applying the MLPA methodology to tumor samples.

**Findings:**

DNA samples from 48 unaffected individuals and 145 breast cancer patients were used to evaluate 11 self-designed MLPA probes and determine the cut-off values for CNV, before applying the MLPA probes to normalize the target probes in a cohort of affected individuals. To test the calculation strategy, three probes were designed to cover regions in Regulator of G-protein Signaling 8 (*RGS8*), which we previously have identified as being affected by allelic imbalance by LOH analysis across *RGS8 *in the cohort comprising 145 breast tumors. Agreement between the LOH results and the results obtained by each of the three MLPA probes in *RGS8 *was found for 64%, 73%, and 91%, of the analyzed samples, respectively.

**Conclusion:**

Here, we present a straightforward method, based upon the normalization pattern in both unaffected and affected individuals, to evaluate self-designed reference probes and to calculate CNV for the MLPA assay with specific focus on the difficulties when analyzing tumor DNA.

## Background

Copy number variations (CNV), including deletions or duplications of whole chromosomes and minor fragments down to single exons are implicated in the onset and risk of human congenital and somatic diseases [1]. The Multiplex Ligation-dependent Probe Amplification (MLPA) is widely used both in research and clinical laboratories and is a promising methodology to assess CNV [2]. MLPA assays provide highly detailed information on CNV, from chromosome aneuploidy down to single exons. The methodology is fast and straightforward to perform, standard laboratory equipment is used, and a limited amount of DNA (20 ng/analysis) is necessary to provide high quality and reproducible results. Ligation-dependent PCR amplification was first described by Hsuih et al. [3] further developed by Schouten et al. [4] and a growing number of predesigned gene specific MLPA assays is commercially available from MRC Holland http://www.mrc-holland.com. The MLPA methodology is versatile and can be applied to different analyses as assessment of genomic copy number variations (CNV), DNA methylation status of specific loci (MS-MLPA), evaluation of chromosomal break points, CGH results, and gene expression profiles (RT-MLPA) [5-8]. The advantage is that multiple loci can be analyzed simultaneously and software is available to perform the tedious calculations.

Current research involving MLPA is mainly based on the commercially available MLPA kits targeting specific genes (MRC-Holland, Amsterdam, The Netherlands), which significantly shorten the optimization time and facilitates data analysis, as specific software is available to analyze the results.

However, the increasing demand for new gene specific assays makes it necessary to self-design individual probes for the analysis [9]. Establishment of CNV is based upon results obtained from reference probes, which are included in each individual assay and used to normalize the values obtained from the target genes. Specific care has to be taken when analyzing tumor genomes, as some of these reference genes may be located in regions affected by cancer specific CNV [10-12].

Here, we describe the evaluation of self-designed reference probes, using a cohort of unaffected and affected individuals, to determine the quality and the cut-off values determining CNV, before applying and using the reference probes to normalize self-designed target probes in tumor samples. To evaluate our calculation strategy we aimed to determine CNV in Regulator of G-protein Signaling 8 (*RGS8*), which previously has been identified as allelic imbalance by a combination of loss of heterozygosity (LOH) analysis and PCR mapping (unpublished results).

### Samples

Breast tumor DNA was isolated from 145 women diagnosed with unilateral primary breast cancer. The cohort was collected between August 1992 and January 1994 and described in [13]. DNA isolated from peripheral blood was obtained from healthy Danish medical students in their first year of University. All DNA was isolated according to [14].

The respective local ethical committees have approved the use of these samples.

### Probe design and MLPA assay

Probes for the MLPA assays were designed according to "Improved synthetic probe design protocol" available on http://www.mrc-holland.com, and the guidelines were followed carefully.

Eleven probes were synthesized at 40 nmol synthesis scale and purchased from TAG Copenhagen A/S (Copenhagen, Denmark). FAM-labeled universal MLPA primers were used to enable simultaneous probe amplification. Probe mixes, used for further analysis, consisted of 0.8-1.6 μl of each 1 μM probe solution in a total volume of 400 μl. Primer sequences are listed in Table 1.

**Table 1:** The set of synthetic oligonucleotide probes used in the MLPA assay.

Probe name	Location	Length (bp)	Left probe hybridizing sequence	Right probe hybridizing sequence
ARX	Xp21.3	83	CGAATGCCGGGCTGATGAAAG	CTGGGTGTCGGAACACTGCC
AFAP1L1a	5q33.1	90	CCTACACTAACACATGATGAAAACC	TCAATATGTCCTCAGGTGGTACC
ACTR1A	10q24.32	102	GGATGAGTTAATTCACACAGCTTTG	TCAGAGCCCTCATGCAGCCTCTTGTAAGCAGATAG
AFAP1L1b	5q33.1	114	GAGACAAGACCTGATCATCTGATCACACTT	GTGCCAACTTGATTCATATTGGGCATTACTAACAACCCCTGG
CFL2	14q13.2	132	ctgacCGTGCTGCCATATCACTAAAATAGGCTTGCCAAGG	CAGGTGAGGTGTATGAATGCTCAAGCCTCACAGAACTGCAATCAAGTGCC
P1	1q25.3	106	gctaCTCCAGAGCAAATAAGCATGG	ACCTCAAGAATGAATTGATGTACCAGTTGGAgactgact
P2	1q25.3	122	CAAGAAGACCAGGTCAACTGCAAAACTGGT	CTCTAAGGCCCATAGGATCTTTGAGGAGTTTGTGGATGTGCAGGCTCCAC
P3	1q25.3	127	gactgactgactCCGTAAATGAACGTATGCTGAACATG	ATCCGTCAGATCTCTAGACCCTCAGCTgactgactgactgactgact
Fr. 14	1q25.3	101	GAGGACGTGAACCAGGACCGTAAGGA	CATGAGCCAGAGCAGTGGTAGCGAGGATGGAGG
Fr. 31	1q25.3	107	TGGGCAGATTCCTTTGATGTGCTTC	TCTCTCATAAGTGTAAGTAGAATTCAGGTCGTATCATGAC
Fr. 32	1q25.3	119	gactGACCTGCGTTCTTGATTTTGACTCAGCG	TTGTTACTAAGTAGTTGGGTGGTCTTGACAGAAACTCgactgact

Upper case correspond to the left and right hybridizing sequence of each probe, whereas lower case correspond to the stuffer sequences used to increase the size of the probe; underlined probes concern the reference probes analyzed in this study.

The MLPA protocol (MLPA^®^DNA protocol) was performed using the instructions provided by the company http://www.mrc-holland.com and initially described by Schouten et al. [4].

The MLPA assay was performed using sets of self-designed probes and the EK5 SALSA MLPA reagents kit purchased from MRC-Holland (Amsterdam, The Netherlands). Briefly, 10 ng of genomic DNA in a volume of 5 μl was denatured for 5 min at 98°C, cooled and hybridized with MLPA probes (1.5 μl/sample) in the presence of SALSA MLPA buffer (1.5 μl/sample) for 16 h at 60°C. Ligation reaction was performed for 15 min at 54°C by adding 32 μl of ligation mixture (3 μl SALSA Ligase buffers A and B, respectively, 25 μl demineralized water, and 1 μl SALSA Ligase-65/sample) followed by heating to 95°C for 5 min. PCR amplification of the ligation product was performed according to the standard protocol. Briefly, 5 μl of the ligation product was added to the mixture consisting of (15.75 μl demineralized water, 2 μl SALSA-PCR buffer, 1 μl SALSA-enzyme dilution buffer, 1 μl SALSA FAM PCR primers and 0.25 μl SALSA polymerase/sample) while the samples were left at 4°C or on ice. The samples were pre-heated to 60°C for 20 s, followed by 35 cycles of 95°C for 30 s, 60°C for 40 s, 72°C for 1 min, and a final extension for 20 min at 72°C. From each PCR reaction, 1.0 μl product was mixed with 9 μl of deionized formamide (Amresco, Solon, Ohio) and 0.3 μl of Genescan-Rox 500 size standard (Applied Biosystems, Foster City, CA, USA) and all fragments were separated by capillary electrophoresis on the ABI 3130XL Genetic Analyzer (Applied Biosystems, Foster City, CA, USA).

### Evaluation of reference probes

Ten DNA samples obtained from unaffected individuals were analyzed in a pilot study to evaluate the general peak pattern of the reference probes in a multiplex set-up.

To secure that the self-designed reference probes were normally distributed, they were evaluated using DNA samples from 48 unaffected and 145 affected individuals. Following the MLPA analysis, all fragment-specific peaks, obtained from capillary electrophoresis, corresponding to specific probes were identified by GeneMapper™ (Applied Biosystems, Foster City, CA, USA) applying the Amplified Fragment Length Polymorphism (AFLP) analysis method. It should be taken into consideration that the AFLP algorithm automatically normalizes the peak heights and that this option should be turned off (recommended by Applied Biosystems). Data of peak height and fragment length were imported into a Microsoft Excel spreadsheet for further analysis.

Peak heights of the reference probes were analyzed in a Gaussian Q-Q plot and in a histogram, where all reference probe peaks have to show the same normalization pattern in both affected and unaffected individuals; otherwise, the probe is omitted from further studies 1. If the samples were overloaded, dilution of the MLPA product should be avoided as it may affect the final result. Instead, we suggest changing the injection time or voltage according to recommendations from MRC Holland. If the problem persists, the volume of probes in the probe mix can be decreased as well as the MLPA product before the capillary electrophoresis. The important issue is to treat all samples from unaffected and affected individuals in exactly the same way throughout the MLPA analysis.

### Normalization of MLPA probes and CNV calculations

CNV was established in the tumor samples by normalization of the target probe to all reference probes present in the run. Next, standard deviation and mean value of the probe of interest were calculated from the cohort of unaffected individuals to establish the z score in the tumor samples. Normalized peak height ratios for each target probe in both tumor samples and unaffected individuals were calculated as:

, where *a *is the peak height of the target probe and *C*_*1*-n _is the peak height of each reference probe *1 - n*. In this case the normalization of the probe height takes into account the specific peak profile and the ratio of the reference probe in the sample. When assessing the quantitative value of each probe, both the peak height and the peak area can be used. We experienced that the peak height provided the most consistent results (data not shown).

Since the normalized peak heights were normally distributed, the z score of normalized peak heights (*x*) in tumor samples was found as , where *μ *and *σ *are the estimated mean and standard deviation of the normalized peak heights for the population of unaffected individuals. To determine CNV for each probe, the distribution of normalized peak heights can be determined from the group of unaffected individuals and the cut-off values determined as fractils from the normal distribution. When the z score was calculated, the cut-off values could be determined from the standard normal distribution, in which +/- 1.96 is equivalent to a 5% cut-off value.

Moreover, all probes in a region analyzed together can be evaluated in combination to provide an overall measure of gain or loss. If there are *k *probes in the region, and an individual has the probe z scores *z*_1_,...,*z*_*k*_, the combined z score is . Under the null hypothesis that *z *_1_,...,*z*_*k *_are standard normal distributed, the *z*' is standard normally distributed too and can be evaluated in the same way as the z scores for each probe.

The Microsoft Office Excell (version 2007) and JMP^® ^Software were used for analyses.

### Analyses of reference probes for MLPA assay

**Figure 1:**
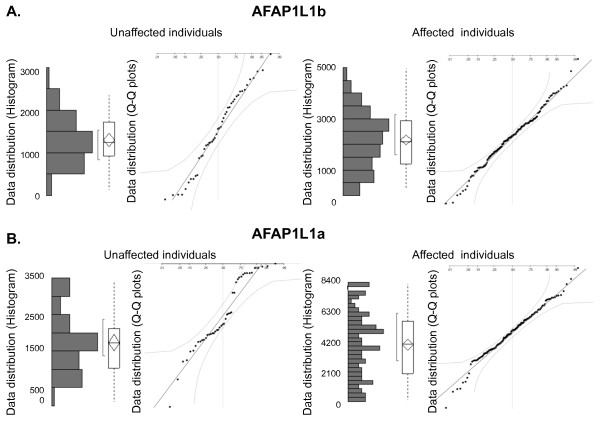
**Establishment of MLPA reference probe quality by statistical analyses of results obtained from cohorts comprising unaffected and affected individuals, respectively.** To ensure a normal distribution of the obtained data for the reference probes AFAP1Lb (A) and AFAP1La (B) in all analyzed cohorts, the z score and Q-Q plots were established based upon data from 48 unaffected individuals and 145 breast tumors (affected individuals). The z score based histograms and Q-Q plots for AFAP1Lb followed a normal distribution for both cohorts, and the reference probe was included in the study (A). Data obtained for the reference probe AFAP1La were not normally distributed as illustrated by the z score based histograms and the Q-Q plots for both cohorts. The probe was excluded from further studies (B).

DNA samples from 48 unaffected individuals and 145 breast tumors were successfully analyzed using 11 self-designed MLPA probes. The peak heights of all five reference probes (*ARX, AFAP1L1a, AFAP1L1b, ACTR1A, CFL2*) were normalized to each other, the z score was calculated and further evaluated in a Q-Q plot to verify the distribution. Based on these analyses, the *ARX, AFAP1L1a *and *ACTR1A *reference probes did not meet the requirements as they varied significantly in samples from both cohorts. *AFAP1L1b *and *CFL2 *showed no significant variation between unaffected and affected individuals and were included in the following CNV analyses. An example of the evaluation in a Gaussian Q-Q plot conducted on the analyzed reference probes is shown in figure 1.

### Validation of CNV by *RGS8 *specific MLPA assay

**Figure 2:**
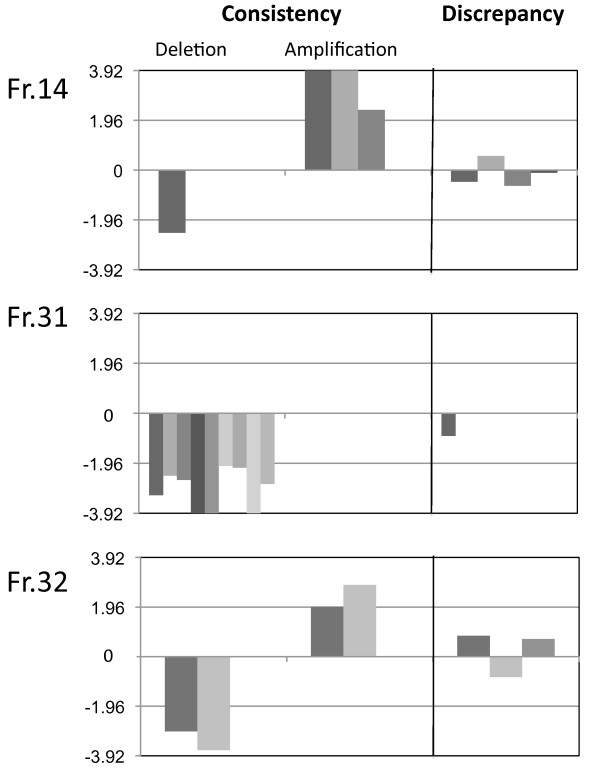
**Consistency and discrepancy between the RGS8 MLPA results and PCR deletion mapping of the same region. ** Each column represents the rate of copy number variation based upon MLPA analysis of 11 tumor samples, in which RGS8 intragenic deletions previously have been established by LOH analysis followed by PCR mapping. For each of the three fragments 14, 31, and 32 agreements between the two methods are illustrated by the rate of deletion and amplification obtained by MLPA for individual samples. Cases with no CNV and agreement between the two methods comprise 3 (fr. 14), 1 (fr. 31) and 4 (fr. 32) samples, are not included in figure. The 95% CI from the normal distribution was used to set the cut-off value for amplification above 1.96 and for the deletion below -1.96.

Three MLPA self-designed target probes were included in a MLPA study to validate the CNV calculation method. All probes were located within specific regions of *RGS8*, which previously has been identified as being affected by allelic imbalance by detailed LOH analysis in breast cancer samples (manuscript in preparation). Exact location of the affected regions was performed by PCR amplification of overlapping fragments throughout the affected region. None of these methods provide unambiguous CNV results. Previously analyzed reference probes AFAP1L1b and CFL2 were included to normalize the data. Eleven breast tumor samples, presenting either single or multiple allelic imbalances within the probe regions, were analyzed. MLPA analyses were conducted as described above. Based on the MLPA results, allelic imbalance of the fr.14 probe were confirmed in 64% (7/11), for the fr.31 probe in 91% (10/11), and for the fr.32 probe in 73% (8/11) (figure 2). Cases with no CNV and agreement between the two methods comprise 3 (fr. 14), 1 (fr. 31) and 4 (fr. 32) samples, are not included in figure 2. 

Our results strongly indicate that the MLPA probes should be designed using exon sequences as templates. The two intron-derived reference probes AFAP1L1a and ACTR1A could not be successfully analyzed due to high rate of variation between unaffected and affected individuals. In contrast, the exon specific AFAP1L1b reference probe presented normal distribution in both unaffected and affected samples enabling to validate MLPA data. The same issue might concern the set of synthetic probes designed to validate previously identified allelic imbalances within the *RGS8 *in breast tumor samples. The target-specific probes in the *RGS8 *except the fr.31 probe were designed in non-coding parts of the gene, due to specific unsuitable sequence composition in the target exons. The number of confirmed deletions was relatively lower for these intron - based probes when compared to fr.31. Different repeated sequences as SINE, LINE or Alu repeats may interfere with the probe hybridization lowering the specificity. Likewise, the presence of an yet uncharacterized single nucleotide polymorphism (SNP) may prevent hybridization or ligation of the probes [15]. Another possible reason for discrepancy in validating CNV in breast cancer by MLPA might be due to non-microdissected tumor DNA.

In conclusion, our approach using a group of unaffected individuals to assess the quality of reference probes, and obtain cut-off values for CNV is to our knowledge new, and a solid supplement to existing analysis software. The aim has been to present simple calculations to facilitate the use of the versatile MLPA methodology especially in cancer research.

## Competing interests

The authors declare that they have no competing interests.

## Authors' contributions

KP participated in the design of the study, performed MLPA analyses, participated in developing the algorithms and statistical analysis, and drafted the manuscript. EW participated in the design of the study, performed the MLPA and *RGS8 *analysis, participated in developing the algorithm and drafted the manuscript. BEM developed the algorithms and the statistical analysis. JO collected the patient cohort and drafted the manuscript. LLH participated in the design of the study, coordination of the study and drafted the manuscript.

All authors have read and approved the final manuscript.
